# Construction of Sensory Evaluation System of Purple Sweet Potato Rice Steamed Sponge Cake Based on Fuzzy Mathematics

**DOI:** 10.3390/foods13213527

**Published:** 2024-11-04

**Authors:** Hongyan Cai, Yanting Liu, Weiping Jin, Fang Li, Xuan Chen, Guoyan Yang, Wangyang Shen

**Affiliations:** 1 School of Food Science and Engineering, Wuhan Polytechnic University, Wuhan 430023, China; l130197yt@163.com (Y.L.); jwpacademic@outlook.com (W.J.); lifang728@126.com (F.L.); chenxuan_1986.163@163.com (X.C.); yang-guo-yan@163.com (G.Y.); whwangyangshen@126.com (W.S.); 2 Key Laboratory for Deep Processing of Major Grain and Oil, Ministry of Education, Wuhan 430023, China

**Keywords:** fuzzy binary comparison, sensory evaluation system, fuzzy mathematics

## Abstract

This paper presents the establishment of a sensory evaluation system for purple sweet potato rice steamed sponge cake (PSPRSSC) utilizing fuzzy mathematics. Initially, eight key sensory evaluation indices were identified through expert consultations. These indices were subsequently prioritized using fuzzy binary comparison, resulting in the following order: aroma, taste, crust color, elasticity, viscosity, chewiness, hardness, and brightness. The corresponding weight values assigned to each index were 23%, 18%, 16%, 13%, 12%, 8%, 6%, and 4%, respectively. Notably, aroma was found to have a more significant impact than visual attributes in the sensory evaluation of PSPRSSC. Based on this prioritization, comprehensive sensory evaluation criteria for PSPRSSC were formulated. Verification tests confirmed the efficacy of the proposed evaluation system. This study provides critical data and theoretical support for the sensory quality assessment of PSPRSSC, thereby facilitating its industrialization and enhancing its market viability.

## 1. Introduction

Rice steamed sponge cake, a traditional Chinese food with a long history, is renowned for its unique flavor and nutritional value [1]. Characterized by its honeycomb structure, soft texture, and notable elasticity, this delicacy also possesses a distinct fermented aroma that facilitates easy digestion [2]. As living standards continue to rise, consumers are increasingly seeking greater variety and enhanced nutritional balance in rice steamed sponge cake, reflecting a shift towards more diverse and health-conscious preferences.

Purple sweet potato (Ipomoea batatas) is a special kind of sweet potato that not only contains nutrients such as starch, dietary fiber, protein, and the microelements of a regular sweet potato, but is also rich in anthocyanins, a group of flavonoids with a benzopyran structure [3,4]. Research indicates that anthocyanins contribute to various health effects, such as antioxidative, antiobesity, immunomodulatory, antiaging, antihyperglycemic, hepatoprotective, antimicrobial, and cardiovascular effects [4,5]. Given its diverse health benefits, appealing color, and distinctive flavor, purple sweet potato serves as an excellent raw material for processed food products. Consequently, it has garnered increasing attention from food researchers in recent years.

Purple sweet potato rice steamed sponge cake (PSPRSSC) is made from purple sweet potato flour and rice flour, which undergo a series of processes including beating, fermentation, and steaming. The resulting cake features a distinctive honeycomb structure and a soft, tender texture, complemented by the unique and pleasant flavor of purple sweet potato. Notably, PSPRSSC retains a significant portion of the nutrients found in purple sweet potato, making it a valuable addition to the category of deep-processed sweet potato products. This product addresses the limitations of traditional rice steamed sponge cakes, which are often characterized by a monotonous taste, uniform color, and nutritional imbalance. The development of PSPRSSC is significant not only for the introduction of new food products but also as a novel application of purple sweet potato in the food field. The addition of purple sweet potato into rice food products significantly enhances their nutritional profile and color, and the physicochemical properties of the raw material [6]. Specifically, this addition increases the content of total dietary fiber and anthocyanins while reducing the level of amylose. The high dietary fiber content in purple sweet potato can influence the texture and taste of the resulting product. Furthermore, the anthocyanins contribute to altering the product’s color. Variations in the content and proportion of starch, amylose, and amylopectin within the blended powder can affect the gelatinization and thermodynamic properties of the composite flours, subsequently impacting the texture and flavor of the final product [7]. Due to the distinct properties of purple sweet potato compared to traditional rice, the processing techniques and quality characteristics of purple sweet potato rice steamed sponge cake (PSPRSSC) will differ from those of conventional rice steamed sponge cake. Therefore, it is essential to establish a sensory evaluation system for PSPRSSC to facilitate further research into determining the optimal technological parameters for its production. There are considerable differences in sensory qualities between PSPRSSC and traditional rice steamed sponge cake, particularly in terms of appearance, aroma, taste, and internal structure. For instance, while traditional rice steamed sponge cakes are typically white and require a higher degree of whiteness [8], PSPRSSC displays a vibrant purple hue. Consequently, the existing sensory evaluation system for rice steamed sponge cake is inadequate for assessing PSPRSSC. Therefore, it is essential to establish a dedicated sensory evaluation system specifically for this innovative product.

The construction of a sensory evaluation system involves several key components, including the determination of sensory evaluation indices for food, the establishment of standards, and the assignment of corresponding grade scores. The selection of these sensory evaluation indices is foundational and critical to the development of an effective sensory evaluation system. Fuzzy mathematics is a methodology that quantifies uncertain factors through mathematical statistics, making it particularly suitable for food sensory evaluation, where many indices are inherently fuzzy and uncertain. By employing fuzzy mathematics, researchers can mitigate the influence of human subjectivity, thereby enhancing the objectivity and reliability of sensory evaluations [9]. Numerous scholars have investigated the construction of sensory evaluation systems for a variety of food products using fuzzy logic. Examples of such research include studies on bread made from millet-based composite flours, beer preferences among Korean consumers, sensory characteristics of Korla pears from different orchards, peanut-sprout yogurt, steamed bread enhanced with cellulase, kokum drinks, and probiotic whey beverages [10,11,12,13,14,15]. Despite its potential benefits, there is a significant lack of research dedicated to the sensory evaluation system specifically for purple sweet potato rice steamed sponge cake (PSPRSSC). Addressing this research gap provides a valuable opportunity for further investigation and development in the sensory assessment of this innovative food product. This exploration could lead to the enhanced understanding and optimization of PSPRSSC, ultimately contributing to its acceptance and success in the food market.

Fuzzy binary comparison is a method within fuzzy mathematics that facilitates the determination of weights based on natural language and cognitive processes, utilizing the complementary definition of fuzzy sets. This approach effectively addresses the challenges associated with unstructured decision-making and is particularly valuable in establishing the weights of sensory evaluation indices through rigorous reasoning [16]. By employing fuzzy binary comparison, researchers can significantly diminish the variability and ambiguity often encountered in food sensory evaluation systems, thereby enabling a process of “fuzzy opinion concentration”. This method enhances the clarity and consistency of sensory evaluations, ultimately contributing to more reliable and valid outcomes in the assessment of food products.

Currently, there is an absence of established quality evaluation standards for PSPRSSC, which significantly hinders both the investigation of technological formulas and the efficiency of industrial production. To advance this field, it is crucial to identify the sensory quality characteristics of PSPRSSC, develop appropriate evaluation methods, and establish a scientifically rigorous sensory evaluation system. Addressing these foundational issues is essential for the progress of research and development in this area. Drawing upon fuzzy mathematical theory, this study constructed a sensory evaluation system for PSPRSSC utilizing fuzzy binary comparison and expert consultation. The sensory evaluation indices were carefully selected, and their corresponding weight distributions were determined. Furthermore, appropriate terms and parameters were established to provide a comprehensive description of the sensory evaluation criteria for PSPRSSC. Ultimately, the validity of the sensory evaluation system was confirmed. This research provides a basis for the evaluation of the sensory quality of PSPRSSC, a judgment method for the optimization of product formulation and process, and a reference for the industrial production of the product.

## 2. Materials and Methods

### 2.1. Materials

The purple sweet potato flour was procured from Shandong Shengdi Sweet Potato Industry Co., Ltd. (Jining, China), while the rice used in this study was obtained from Yihai Kerry Cereals, Oils, and Foodstuffs Co., Ltd. (Shanghai, China). Additionally, the yeast utilized in the formulation was sourced from Angel Yeast Co., Ltd. (Yichang, China).

### 2.2. Preparation of PSPRSSC

The preparation of PSPRSSC was conducted following the modified method established by [1]. The optimized production process for PSPRSSC, based on preliminary experimental results, was outlined as follows: Raw rice was milled into flour using a cyclone grinder (JXFM110, Shanghai Jiading grain and oil Instrument Co., LTD., Shanghai, China) and subsequently passed through a 100-mesh sieve. A mixture was prepared by combining 15 g of purple sweet potato flour, 85 g of rice flour, 20 g of white granulated sugar, 3 g of yeast, and 115 g of water. The mixture was thoroughly stirred and allowed to ferment in a fermenting box (JXFD 7, Beijing Dongfu Jiuheng Instrument Technology Co., LTD., Beijing, China) at 37 °C for 1 h. Following fermentation, the mixture was transferred into a specialized mold designed for the cake and steamed for 15 min. After steaming, the sample was allowed to cool to room temperature.

### 2.3. Selection of Sensory Evaluation Indices

**Figure 1:**
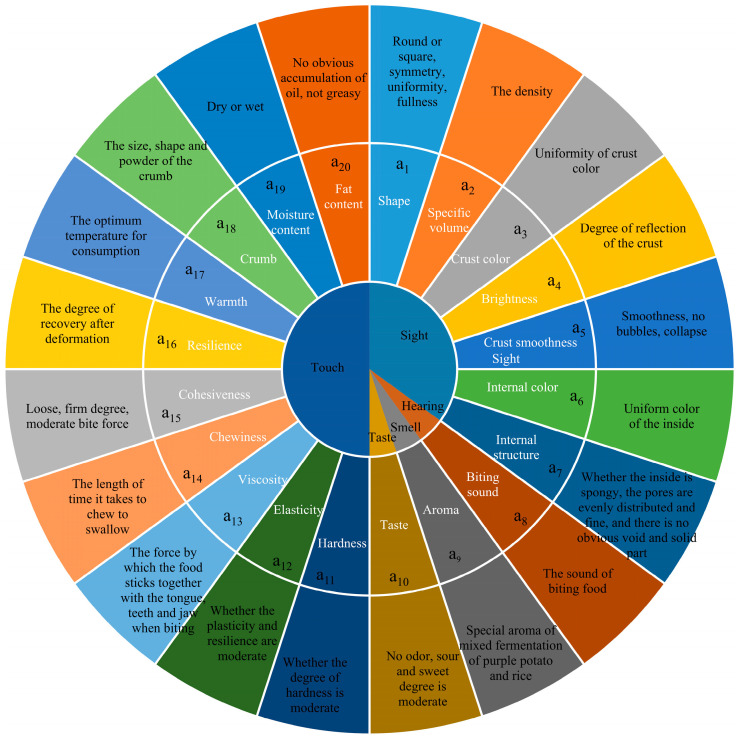
Sensory evaluation indices of PSPRSSC, and a_i_ (i = 1, 2, 3 … 20) is the number of 20 sensory indicators.

The sensory characteristics of food can be categorized into five primary dimensions: appearance, color, texture, flavor, and taste. Appearance encompasses the shape, size, and surface texture of the food product. Color refers to both the external and internal hues of the food. Texture pertains to the tactile sensory qualities, including hardness, viscosity, and elasticity. Flavor primarily relates to the aroma of the food as perceived by the sense of smell. Taste, on the other hand, describes the sensory characteristics associated with flavor, including the sensations of sourness, sweetness, bitterness, spiciness, and saltiness [17]. The sensory quality evaluation indices for PSPRSSC refer to the quality characteristics that can be assessed through the senses of sight, hearing, smell, taste, and touch. While these sensory attributes can be perceived by individuals, they are inherently difficult to measure accurately through physical or chemical means. Numerous indices are associated with the sensory quality evaluation of purple sweet potato rice cake. The selected quality indices have been established based on a comprehensive review and analysis of existing research, alongside relevant studies pertaining to rice steamed sponge cake [18,19,20,21] and the intrinsic quality characteristics of purple sweet potato rice cake. The sensory quality indices of PSPRSSC were systematically classified and defined, prioritizing the selection of indices that possess clear and meaningful descriptions to enhance the robustness and clarity of the evaluation framework. The sensory quality of PSPRSSC was classified based on the five aspects of sight, smell, taste, touch, and hearing, and 20 sensory evaluation indices to be screened were obtained, as illustrated in Figure 1.

### 2.4. Screening Method for Sensory Evaluation Indices

A consultation survey was conducted to evaluate the sensory quality indices of PSPRSSC from an initial pool of 20 quality evaluation indices. The scoring system employed a seven-point scale (1–7 points), where higher scores indicated greater importance. A panel of 10 investigators, all of whom were technical personnel engaged in food research, provided their insights regarding the sensory characteristics of PSPRSSC [22]. Based on the results of this expert consultation survey, preference indices were selected from the original 20 indices to establish a refined set of sensory evaluation criteria. The priority ranking was defined as the ratio of the total score achieved by each group to the maximum possible score of 70 points. This value was maintained with four decimal places for precision.

### 2.5. Priority Ranking Method for Sensory Evaluation of Optimal Indices

Based on the outcomes of the consultation survey, eight preferred indicators were selected from an initial pool of 20 indices. To determine the priority of these eight preferred indices, a fuzzy binary comparison method was employed [23,24]. This involved conducting pairwise comparisons, where each pair of optimal indices was assigned a score on a scale of 1 to 10, reflecting their relative advantages and disadvantages (Table 1). A total of 18 experts participated in the survey, all of whom were professionals engaged in food research and development for a long time. The preference selection ratios were then statistically calculated, resulting in the formation of a preference selection ratio matrix. For instance, if color serves as the contrasting element and brightness as the standard element, a total score for color comparison was assigned by 18 sensory evaluators. If the resulting score is 102, the preference selection ratio of color to brightness can be calculated as U_12_ = 102/180 = 0.5667. Consequently, the preference ratio of brightness to color is U_21_ = 1 − U_12_ = 0.4333. The preference selection ratios for all indices were statistically calculated, resulting in the formation of a preference selection ratio matrix that adhered to the condition, which satisfied U_ij_ + U_ji_ = 1 (i = 1, 2, 3 … 8, j = 1, 2, 3, … 8).

Subsequently, a threshold calculation ranking method was applied. This method involved identifying the minimum value from each non-diagonal element of the preference selection ratio matrix, followed by determining the maximum value among these minimums. The index corresponding to the row containing this maximum value was deemed the most superior. The row and column associated with this superior index were then removed from the matrix, and the process was iteratively repeated to establish the priority order of each indicator.

After conducting pairwise comparisons for all indicators, the results were organized using a fuzzy priority relation order method, leading to the final ranking of the eight preferred indicators. A total of 18 experts participated in the consultation survey, all of whom were seasoned professionals engaged in food research and development.

### 2.6. Determination of the Weight of Optimal Indices in Sensory Evaluation

The total score for the eight sensory evaluation indices was established at 100 points [19]. To assess the importance of each index, five food research technicians were invited to assign scores based on their evaluations. In accordance with the results from the fuzzy binary comparison priority ranking, the score assigned to any lower-priority index was constrained to ensure it did not exceed that of a higher-priority index. To determine the weight distribution for each index, the average scores from the technicians were calculated and subsequently normalized. This normalization process allowed for a consistent representation of the relative importance of each sensory evaluation index within the overall framework, facilitating a clearer understanding of their respective contributions to the sensory evaluation of the product.

### 2.7. Validation of Sensory Evaluation System

As described in Section 2.2 concerning the preparation of purple sweet potato rice steamed sponge cake (PSPRSSC), four samples of rice cakes were created with the varying sugar content levels of 5%, 10%, 15%, and 20%. After undergoing the steaming process, the samples were allowed to cool at room temperature for 15 min. Subsequently, six evaluators employed the newly established sensory rating table to assess the samples, scoring them and calculating the average score for each sample.

### 2.8. Statistical Analysis

The data were processed using Excel software (Microsoft Excel 2016 4.3.5.10), and statistical analysis was conducted employing an analysis of variance (ANOVA). This method allowed for the evaluation of differences among group means and the assessment of variability within the data. ANOVA was utilized to determine whether there were statistically significant differences in the sensory evaluation indices, providing insights into the effects of different factors on the sensory quality of the product.

## 3. Results and Discussion

### 3.1. Screening of Sensory Evaluation Indices 

The results of the sensory quality evaluation indices for PSPRSSC are presented in Table 2. The average scores and variances for the preferred indices were calculated based on the scoring statistics. Higher average scores indicate the greater importance of the respective indices; thus, indices with larger mean values should be prioritized by producers. According to the data in Table 1, the mean values of the sensory evaluation indices for PSPRSSC are ranked as follows: 1. taste; 2. aroma; 3. crust color; 4. viscosity and chewability; 5. elasticity; 6. hardness; 7. brightness; 8. crust smoothness; 9. interior structure; 10. crumb and shape; 11. specific volume; 12. resilience and warmth; 13. moisture content; 14. interior color; 15. cohesiveness; 16. biting sound; and 17. fat content. The analysis revealed that taste, aroma, and crust color received the highest average scores, indicating their critical role in the sensory evaluation of PSPRSSC. Furthermore, a smaller variance suggests greater stability in the evaluation of these indices. The analysis of variance confirmed that the taste, aroma, and crust color indices exhibited strong stability, reflecting a consensus among evaluators regarding their importance. For instance, in the evaluation of the taste index, over half of the group of ten participants regarded it as the most important attribute. Conversely, for indices such as fat content and biting sound, there was considerable variability in their perceived importance, resulting in larger variances and less agreement among evaluators. The eight optimal indices encompass four sensory dimensions: sight (crust color and brightness), smell (aroma), taste (taste), and touch (hardness, elasticity, viscosity, and chewiness). This comprehensive selection effectively addresses all sensory dimensions relevant to food sensory evaluation. Consequently, it can be concluded that these eight optimal indices provide a thorough representation of the sensory properties of PSPRSSC, ensuring a holistic assessment of its sensory qualities.

### 3.2. Priority Ranking of Sensory Evaluation Indices of PSPRSSC

The eight optimal indices were renumbered as *bi* (where *i* = 1,2,3, …, 8) according to their order of occurrence in Table 2. The domain U = {U_1_, U_2_, … U_8_} represents the indices of PSPRSSC. A fuzzy binary comparison approach was employed to establish a fuzzy priority relationship among these indices, allowing for the calculation of total scores and priority selection ratios based on the scores obtained from each group of comparative evaluations. The statistical results of the fuzzy binary comparison are presented in Table 3. Priority selection reflects the relative importance of each index; specifically, a priority selection ratio greater than 0.50 indicates that the comparative element holds a higher priority than the reference element. As evidenced in Table 3, when crust color is considered the contrasting element, it is observed that crust color takes precedence over brightness, hardness, and viscosity. Conversely, aroma, taste, elasticity, and chewiness are deemed more significant than color. The priority matrix for the evaluation indices was constructed based on the priority selection ratios of the eight indicators, as detailed in Table 4. The priority order of each index was determined using a threshold calculation ranking method. For instance, the maximum value among the minimum values in all rows was 0.4667, which means that aroma from row U_3_ was the highest priority indicator. The U_3_ row and U_3_ column were removed to obtain a new matrix (Table 5). Subsequently, the next priority indicator was determined based on this updated matrix, and the priority order of all indicators was established through this iterative process. The resulting priority sequence for the sensory evaluation indices of PSPRSSC was as follows: 1. aroma; 2. taste; 3. crust color; 4. elasticity; 5. viscosity; 6. chewiness; 7. hardness; and 8. brightness. The findings indicate that aroma, taste, and color are critical attributes for the sensory quality of the product, aligning with existing literature reports [25]. 

### 3.3. Determination of Optimal Index Weight of PSPRSSC

The priority order of the eight optimal indices, determined through fuzzy binary comparison, was renumbered as *ci* (where *i* = 1, 2, 3, …8). The results of the weight investigation for the optimal indices in the sensory evaluation of PSPRSSC are presented in Table 6. The average score for each index was approximated, leading to the following weight distribution among the preferred indices: aroma (23%), taste (18%), crust color (16%), elasticity (13%), viscosity (12%), chewiness (8%), hardness (6%), and brightness (4%). The weight distribution indicated that aroma held the highest weight at 23%, which aligned with established sensory perceptions regarding food. Conversely, brightness had the lowest weight at 4%. Notably, aroma was categorized under olfactory senses, whereas brightness pertained to visual perception. This finding suggests that olfactory factors play a more significant role in the sensory evaluation of PSPRSSC compared to visual factors, underscoring the importance of aroma in influencing consumer preferences and perceptions of food quality.

### 3.4. Construction of Sensory Evaluation System of PSPRSSC

The method of assessing the evaluation indices or quality characteristics of a sample using a numerical scale is referred to as the scoring method. The scoring methods and rules for evaluating the quality characteristics of PSPRSSC are essential components of the quality evaluation system. Establishing scientific and rational scoring rules and methods for each evaluation index is crucial for ensuring that the quality evaluation process is conducted smoothly and effectively. Furthermore, it is important to develop a comprehensive quality evaluation system subsequent to determining the quality evaluation indices and their respective weights for PSPRSSC. A full sensory score for PSPRSSC was established at 100 points, with the maximum scores allocated to each preferred index as follows: aroma (23 points), taste (18 points), crust color (16 points), elasticity (13 points), viscosity (12 points), chewiness (8 points), hardness (6 points), and brightness (4 points), in accordance with the weights of the eight optimal indices. To develop a comprehensive sensory evaluation standard for PSPRSSC, it is essential to provide detailed descriptions for each corresponding sensory evaluation index. Quantitative description is a widely utilized methodology in sensory scoring. After assigning specific scores to represent the quality characteristics of an indicator across various stages or degrees of intensity, evaluators assess the samples and determine the corresponding scores based on their subjective perceptions. This scoring process allows for the systematic evaluation of sensory attributes, facilitating a more structured analysis of the quality characteristics.

The descriptions of the sensory indices for PSPRSSC primarily referred to the literature [12,26,27]. By synthesizing and organizing these descriptions, combined with the characteristics of PSPRSSC, the sensory evaluation system of PSPRSSC was established. The specific sensory evaluation descriptions for PSPRSSC are presented in Table 7. This table outlined the attributes and criteria used to assess each sensory index, providing a clear framework for the evaluation process. By standardizing these descriptions, we aim to enhance the consistency and reliability of sensory assessments for PSPRSSC, thereby enabling more accurate evaluations of product quality.

### 3.5. Sensory Evaluation Verification

The sensory evaluation results of PSPRSSC are presented in Table 8, which delineates the differences in sensory scores associated with the varying levels of sugar added. Notably, Sample 4, which contained 20% sugar, achieved the highest sensory score, signifying optimal overall quality. However, a closer examination of the individual indicators within the sensory evaluation framework reveals variability in the quality of the four types of PSPRSSC. For instance, Sample 4 received the highest rating for elasticity, while Sample 1 excelled in hardness. This indicates that the addition of sugar not only enhances the elasticity of PSPRSSC but also diminishes its hardness. Consequently, the sensory evaluation system developed through fuzzy mathematics effectively distinguished the quality of each sensory index of PSPRSSC, enabling a more nuanced understanding of its attributes.

## 4. Conclusions

This study identified eight optimal indices for the sensory evaluation of PSPRSSC through a comprehensive investigation and employed the fuzzy binary comparison method to establish their priority order. Based on this analysis, we determined the weight distribution for each optimal index and developed a sensory evaluation system for PSPRSSC. The eight selected indices corresponded to four sensory dimensions: sight (crust color and brightness), smell (aroma), taste (taste), and touch (hardness, elasticity, viscosity, and chewiness). The priority order of these indices, as determined by the fuzzy priority ranking method, was as follows: aroma, taste, crust color, elasticity, viscosity, chewiness, hardness, and brightness. The respective weights for these indices were 23%, 18%, 16%, 13%, 12%, 8%, 6%, and 4%. This observation suggests that olfactory factors play a more prominent role than visual factors in the sensory evaluation system of PSPRSSC. Building on this insight, a comprehensive sensory scoring system for PSPRSSC was ultimately established. Utilizing the newly developed sensory evaluation framework, sensory assessments were conducted on samples with varying sugar levels, revealing significant differences in sensory scores (*p* < 0.05). Notably, the sample with 20% added sugar received the highest sensory score. The sensory evaluation system, formulated through fuzzy mathematics, demonstrates significant effectiveness in assessing the sensory quality of PSPRSSC. Furthermore, the establishment of this sensory system provides a valuable reference for optimizing the formulation and production processes of PSPRSSC in the future.

The findings of this study contribute valuable insights into the sensory quality assessment of PSPRSSC, providing foundational data for its development and supporting the industrialization of PSPRSSC. The establishment of a sensory evaluation system utilizing fuzzy mathematics marks a significant advancement in the quality assessment of PSPRSSC. This approach effectively integrates qualitative sensory attributes with quantitative analysis, facilitating a more nuanced understanding of consumer preferences. By leveraging fuzzy logic, the system accommodates the inherent subjectivity of sensory evaluations, enabling a comprehensive analysis that captures the variability of human perception. This methodology not only enhances the reliability of sensory assessments but also aids in optimizing product formulations, ultimately contributing to improved consumer satisfaction and market competitiveness. Fuzzy mathematics plays a critical role in the sensory evaluation system by quantifying sensory indices, addressing the uncertainties associated with these indices, and selecting those most suitable for PSPRSSC. Intelligent sensory evaluation offers certain advantages in comparison with artificial sensory evaluation, as it employs advanced equipment to mitigate the subjectivity inherent in human assessments, thereby providing standardized and repeatable evaluation results. Looking ahead, the integration of an artificial sensory evaluation system with intelligent sensory technologies such as texture analyzers, gas chromatography–mass spectrometry (GC–MS), electronic noses, and electronic tongues will enable more precise and comprehensive evaluations of texture and flavor for PSPRSSC. This consideration is crucial for future product development and quality assessment efforts.

Despite the inherent limitations in comparability and repeatability associated with sensory evaluation methods, they remain a predominant approach for assessing food quality. Currently, no alternative method can fully supplant the significance of sensory evaluation in the realm of food quality assessment. Objective evaluation, which employs specific instruments or methodologies to quantify quality traits that can be perceived through human senses, offers results that typically exhibit strong comparability and repeatability. Future research endeavors could utilize the sensory evaluation of PSPRSSC as a reference or benchmark to explore the correlation between objective and sensory evaluation methods. This approach could facilitate a more streamlined and effective evaluation of the product quality of PSPRSSC.

## Figures and Tables

**Table 1:** Scoring criteria of the fuzzy binary comparison of PSPRSSC.

Standard	The Least Important	1 Point~10 Points, Increasing in Importance								The Most Important
Score	1	2	3	4	5	6	7	8	9	10

**Table 2:** The result of the expert survey for the sensory evaluation indices of PSPRSSC.

No.	Items	Score							Total Points	Variance	Priority Rate	Rank
		1	2	3	4	5	6	7				
a_1_	Shape			2	1	5	2		47	1.24	0.671	11
a_2_	Specific volume			2	2	4	2		46	1.04	0.657	13
a_3_	Crust color				2	1	6	1	56	0.84	0.800	3
a_4_	Brightness				3	4	3		50	0.60	0.714	8
a_5_	Crust smoothness				3	5	2		49	0.49	0.700	9
a_6_	Internal color		1	2	2	5			41	1.09	0.586	17
a_7_	Internal structure			1	3	4	1	1	48	1.16	0.686	10
a_8_	Biting sound		3	2	1	2	2		38	2.36	0.543	19
a_9_	Aroma					1	2	7	66	0.44	0.943	2
a_10_	Taste						1	9	69	0.09	0.986	1
a_11_	Hardness				3	3	4		51	0.69	0.729	7
a_12_	Elasticity			1	1	2	6		53	1.01	0.757	6
a_13_	Viscosity				1	3	6		55	0.45	0.786	4
a_14_	Chewiness				2	2	5	1	55	0.85	0.786	4
a_15_	Cohesiveness		1	2	3	4			40	1.00	0.571	18
a_16_	Resilience		1		4	4	1		44	1.04	0.629	14
a_17_	Warmth		1	2	2	2	3		44	1.84	0.629	14
a_18_	Crumb			2	4		3	1	47	1.81	0.671	11
a_19_	Moisture content		2	1	1	4	2		43	2.01	0.614	16
a_20_	Fat content		4		3	3			35	1.65	0.500	20

**Table 3:** Statistics of the fuzzy binary comparison of the sensory evaluation indices of PSPRSSC.

Contrast Element	Standard Element	Score										Total Points	Preferred Selection Ratio
		1	2	3	4	5	6	7	8	9	10		
Crust color	Brightness			2	2	7	2	1	3		1	102	0.5667
	Aroma	1	2	2	2	5	2	2	2			86	0.4778
	Taste	1		5	3	4	1	1	3			85	0.4722
	Hardness		2	2	3	2	3	3	3			95	0.5278
	Elasticity	1	1	4	2	3	2	1	4			89	0.4944
	Viscosity	1		3	2	4	4	2	1	1		93	0.5167
	Chewiness	1	1	5	1	4	3	2	1			82	0.4556
Brightness	Aroma	1	4	4	2	2		2	3			77	0.4278
	Taste	1	4	3	3	2		2	3			78	0.4333
	Hardness		3	4	1	4		5	1			85	0.4722
	Elasticity		4	4	2	3	1	2	2			79	0.4389
	Viscosity		4	5	3	1	1	2	2			76	0.4222
	Chewiness		7	1	2	2	3	1	2			76	0.4222
Aroma	Taste			1	1	7	3	4	1		1	106	0.5889
	Hardness	1	3		4	3	1	2	3	1		91	0.5056
	Elasticity	1	2		3	2	5	3	2			94	0.5222
	Viscosity	1	2		4	3	5	2	1			88	0.4889
	Chewiness	1	1	2	3	5	4	2				84	0.4667
Taste	Hardness	1	1		2	2	2	2	5	3		114	0.6333
	Elasticity	1	1		2	1	2	3	5	3		116	0.6444
	Viscosity	1	1	2		1	3	2	7	1		111	0.6167
	Chewiness	1		2	1	4	2	3	2	3		107	0.5944
Hardness	Elasticity		1		1	11	2	1		1	1	99	0.5500
	Stickness			1	3	11		1	1		1	95	0.5278
	Chewiness		1		4	10	1		1		1	92	0.5111
Elasticity	Viscosity					11	2	1	3		1	108	0.6000
	Chewiness				2	8	2	4	1		1	106	0.5889
Viscosity	Chewiness		1		3	7	2	2	2		1	68	0.3778

**Table 4:** Prior matrix of sensory evaluation indices.

Items	U_1_	U_2_	U_3_	U_4_	U_5_	U_6_	U_7_	U_8_
U_1_	1	0.5667	0.4778	0.4722	0.5278	0.4944	0.5167	0.4556
U_2_	0.4333	1	0.4278	0.4333	0.4722	0.4389	0.4222	0.4222
U_3_	0.5222	0.5722	1	0.5889	0.5056	0.5222	0.4889	0.4667
U_4_	0.5278	0.5667	0.4111	1	0.6333	0.6444	0.6167	0.5944
U_5_	0.4722	0.5278	0.4944	0.3667	1	0.5500	0.5278	0.5111
U_6_	0.5056	0.5611	0.4778	0.3556	0.4500	1	0.6000	0.5889
U_7_	0.4833	0.5778	0.5111	0.3833	0.4722	0.4000	1	0.5611
U_8_	0.5444	0.5778	0.5333	0.4056	0.4889	0.4111	0.4389	1

Note: Items U_1_–U_8_ represent crust color, hardness, aroma, taste, hardness, elasticity, viscosity, and chewability, respectively.

**Table 5:** Prior matrix of sensory evaluation indices after the first preferred indicator was removed.

Items	U_1_	U_2_	U_4_	U_5_	U_6_	U_7_	U_8_
U_1_	1	0.5667	0.4722	0.5278	0.4944	0.5167	0.4556
U_2_	0.4333	1	0.4333	0.4722	0.4389	0.4222	0.4222
U_4_	0.5278	0.5667	1	0.6333	0.6444	0.6167	0.5944
U_5_	0.4722	0.5278	0.3667	1	0.5500	0.5278	0.5111
U_6_	0.5056	0.5611	0.3556	0.4500	1	0.6000	0.5889
U_7_	0.4833	0.5778	0.3833	0.4722	0.4000	1	0.5611
U_8_	0.5444	0.5778	0.4056	0.4889	0.4111	0.4389	1

**Table 6:** The weight values of preferred sensory evaluation indices of PSPRSSC.

Items	Score					Mean	Weight Coefficient/%
	I	II	III	IV	V		
Aroma	22	23	25	20	24	22.8	23
Taste	19	16	20	20	15	18	18
Crust Color	15	15	20	17	14	16.2	16
Elasticity	15	13	10	13	14	13	13
Viscosity	13	12	10	15	12	12.4	12
Chewiness	9	9	7	7	10	8.4	8
Hardness	4	8	5	3	8	5.6	6
Brightness	3	4	3	5	3	3.6	4

**Table 7:** The sensory evaluation criteria of PSPRSSC.

Items	Evaluation Criteria	Score
Aroma (23 points)	There is a special flavor of mixed fermentation of purple potato and rice, and the smell is pure and strong	18–23
	There is a special aroma of mixed fermentation of purple sweet potato and rice, but the smell is light	12–17
	No special smell, no odor	6–11
	The smell is not pure, sour or unpleasant	0–5
Taste (18 points)	There are rice and purple potato fermentation of special taste, taste pure, sweet and sour moderate	13–18
	It has the taste of pure fermented food and the taste is more suitable	7–12
	The fermented flavor of purple potato is light or strong, and the taste is not comfortable	0–6
Crust color (16 points)	Pure color	11–16
	Color dodge	6–10
	Dull in color	0–5
Elasticity (13 points)	Good elasticity, chewy	10–13
	Average elasticity, slightly chewy	5–9
	Poor elasticity, no chewy	0–4
Viscidity (12 points)	Refreshing, sticky, non-stick	9–12
	Sticky but slightly sticky teeth	5–8
	Soft and sticky	0–4
Chewiness (8 points)	Good bite strength when chewing, easy to chew	7–8
	Strong bite, not easy to chew	4–6
	Poor bite strength and slagging or chewing dry when divided	0–3
Hardness (6 points)	Moderate hardness, easy to chew, comfortable taste	4–6
	Very soft or very hard, unpleasant taste	0–3
Brightness (4 points)	The crust is bright	3–4
	The crust is dull	0–2

**Table 8:** The sensory evaluation results of PSPRSSC.

Sample	Aroma (23)	Taste (18)	Crust Color (16)	Elasticity (13)	Viscosity (12)	Chewiness (11)	Hardness (6)	Brightness (4)	Score
1	17.50 ± 1.89 ^a^	11.67 ± 1.70 ^a^	12.50 ± 1.38 ^a^	10.33 ± 1.25 ^b^	8.17 ± 0.90 ^a^	6.67 ± 0.94 ^a^	4.33 ± 0.47 ^c^	3.00 ± 0.58 ^a^	74.00 ± 4.58 ^a^
2	17.83 ± 1.67 ^a^	13.00 ± 1.15 ^ab^	13.17 ± 1.57 ^ab^	11.00 ± 1.00 ^b^	8.33 ± 0.94 ^a^	6.33 ± 0.75 ^ab^	4.17 ± 0.69 ^bc^	3.50 ± 0.50 ^a^	77.67 ± 4.31 ^ab^
3	19.00 ± 1.91 ^ab^	14.17 ± 1.34 ^b^	13.67 ± 1.11 ^bc^	11.33 ± 0.94 ^ab^	9.50 ± 0.50 ^ab^	6.50 ± 0.96 ^b^	4.50 ± 0.76 ^bc^	3.50 ± 0.50 ^a^	82.00 ± 3.56 ^bc^
4	20.50 ± 1.38 ^b^	15.00 ± 0.82 ^b^	13.83 ± 1.34 ^b^	11.33 ± 1.25 ^c^	10.17 ± 1.21 ^b^	7.33 ± 0.75 ^bc^	5.17 ± 0.69 ^ab^	3.50 ± 0.76 ^ab^	86.17 ± 4.26 ^c^

Note: samples note: Samples 1, 2, 3, and 4 represent the sugar addition amounts of 5%, 10%, 15%, and 20%, respectively; different letters in the same column indicate significant differences (*p* < 0.05).

## Data Availability

The original contributions presented in the study are included in the article. Further inquiries can be directed to the corresponding author.
